# Anterior Abdominal Wall Dermatofibrosarcoma Protuberans: A Rare Case

**DOI:** 10.7759/cureus.66627

**Published:** 2024-08-11

**Authors:** Bhushan Shah, Adithya R Vijendra

**Affiliations:** 1 General Surgery, Dr. D. Y. Patil Vidyapeeth, Pune, IND

**Keywords:** dermatofibrosarcoma protuberans, anterior abdominal wall, wide local excision, recurrence, split skin grafting

## Abstract

Dermatofibrosarcoma protuberans is an uncommon, indolently progressive, locally aggressive soft tissue neoplasm that characteristically arises from the dermal and subcutaneous layers. While excision is the primary treatment modality, addressing defects following tumor removal can be challenging, particularly in cases involving the anterior abdominal wall. This publication paper presents a case study of a patient with dermatofibrosarcoma protuberans in the anterior abdominal wall, detailing the surgical approach and subsequent defect repair using skin grafting. We also provide a comprehensive review of the clinical presentation, diagnostic methods, surgical options, and outcomes associated with dermatofibrosarcoma protuberans in this unique location.

## Introduction

Dermatofibrosarcoma protuberans (DFSP) is an uncommon cutaneous soft tissue sarcoma exhibiting a characteristic infiltrative growth pattern, originating within the dermis and subcutaneous tissues [1]. Although DFSP has a low risk of metastasis, it is classified as an intermediate-grade malignancy due to its high likelihood of local recurrence [2]. The cause of DFSP is unclear. About 1% of all soft tissue sarcomas are DFSP, which is often misdiagnosed at first because of its characteristic nodules that appear benign [3]. The primary therapeutic approach for DFSP involves the excision of the tumor with clear surgical margins to mitigate the risk of local tumor recurrence [4]. Reconstruction following excision can vary depending on the size and location of the defect. This publication paper reports a case of anterior abdominal wall DFSP and its surgical management with split-thickness skin grafting.

## Case presentation

A male patient of 73 years came with complaints of a progressively enlarging and painful mass over the anterior chest wall. The mass had been present for more than two years but had demonstrated significant growth in the preceding six months. The patient reported no constitutional symptoms and denied any history of trauma to the affected area. Additionally, the patient’s family history was not particularly noteworthy for malignancies.

**Figure 1:**
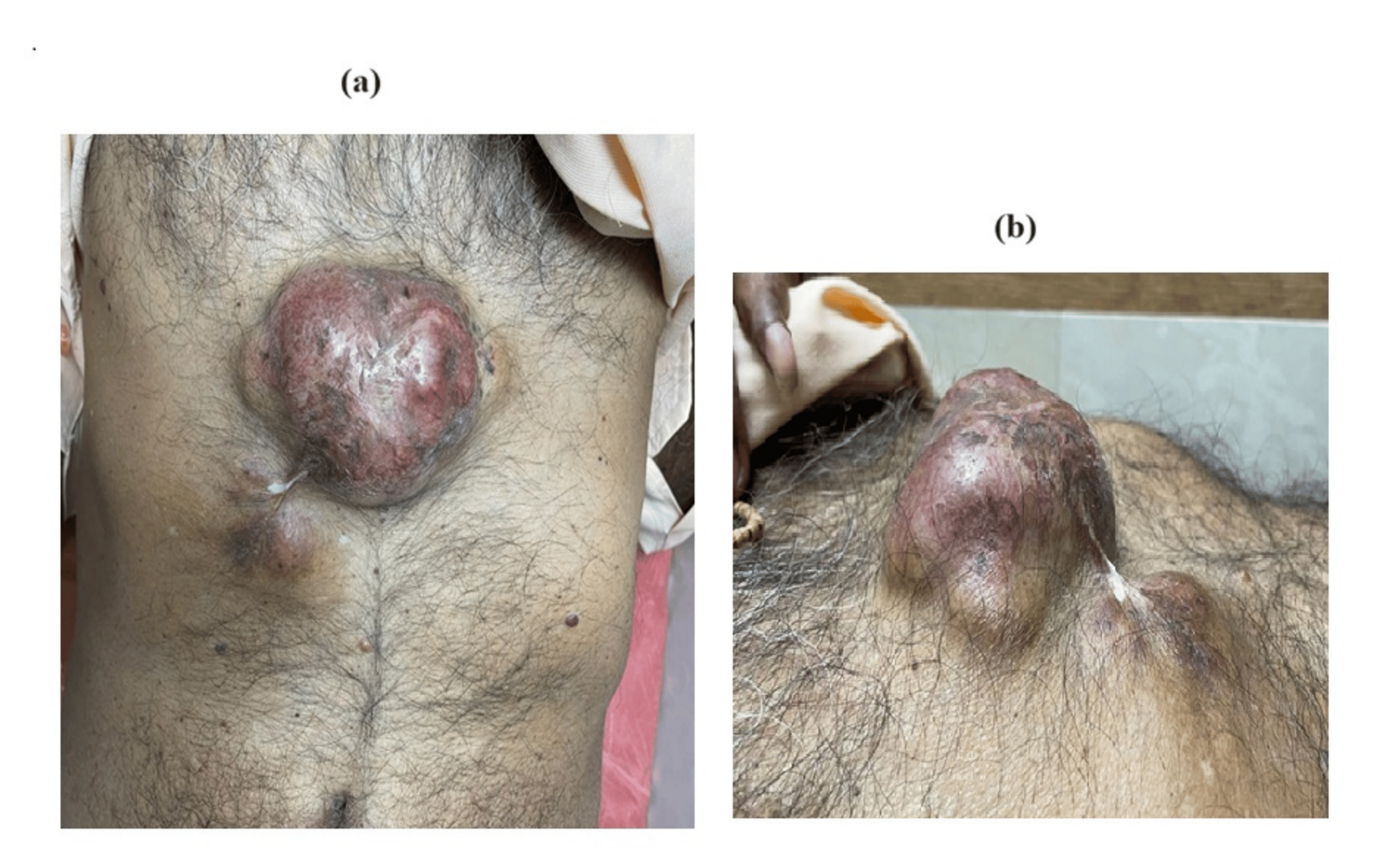
Clinical photo of the patient on presentation (a) Front view of anterior abdominal wall with lesion (b) Side view of the lesion on the anterior abdominal wall

Upon clinical examination, a firm, immobile mass measuring 10 cm x 8 cm was located on the lower chest. The swelling consisted of a smooth exterior, a solid consistency, and adhering skin that overlaid hyperpigmented skin (Figure 1a, 1b).

**Figure 2:**
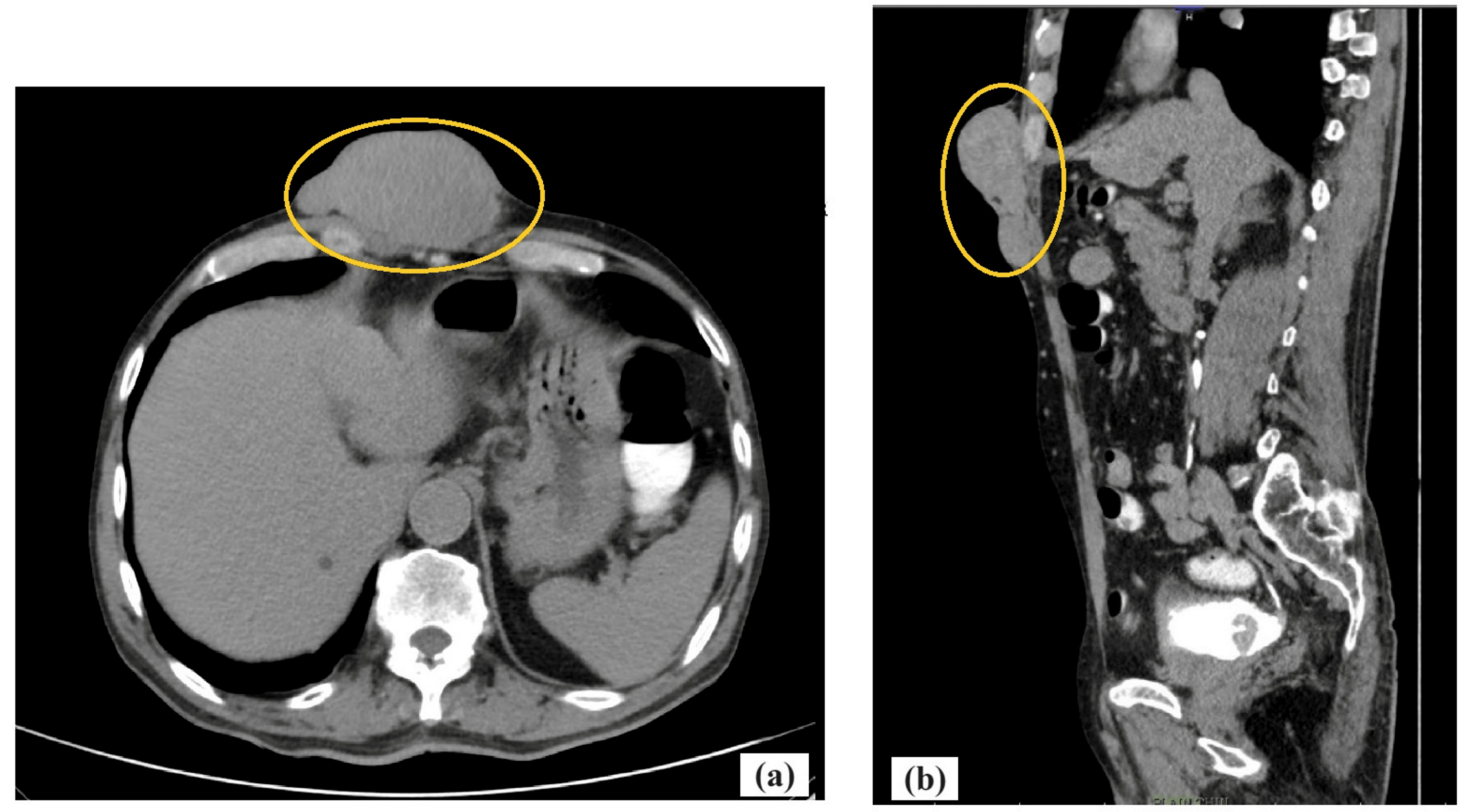
CECT abdomen and pelvis (a) Axial view showing lobulated soft tissue density mass lesion (yellow highlighted part) seen in subcutaneous plane in the anterior abdominal wall in the midline of epigastric region. (b) Sagittal view showing lobulated lesion (yellow highlighted part) with moderate heterogenous enhancement and loss of fat planes with underlying bilateral recti abdominis muscles. CECT - Contrast-enhanced computed tomography

The patient then underwent ultrasonography which revealed a heterogeneously hypoechoic lesion with internal vascularity in subcutaneous plane in the epigastric region. No evidence of deep fascial involvement or regional lymphadenopathy was noted. A CT scan of the thorax and abdomen was done to assess the tumor extent, which reported as lobulated soft tissue density mass lesion of approximately 92x45x74 mm (TRA x AP x CC) seen in subcutaneous plane in anterior abdominal wall in epigastric region with no intraabdominal extension although another lesion with similar morphology is noted of size 39x12 mm in epigastric region below the larger lesion (Figure 2a, 2b).

**Figure 3:**
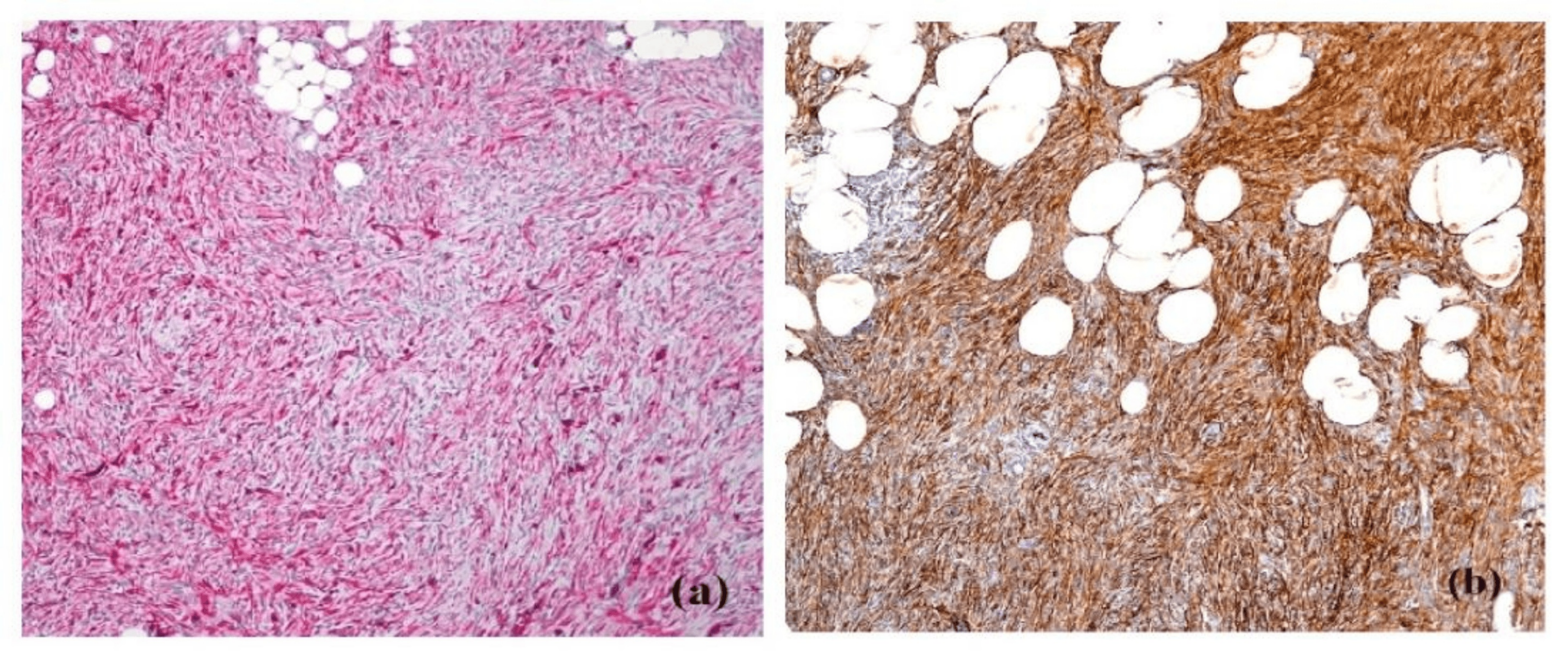
Histopathology images of dermatofibrosarcoma protuberans (a) Hematoxylin and Eosin staining (b) CD34 staining

Microscopic analysis of the tru-cut biopsy sample demonstrated a proliferation of spindle-shaped cells organized in a whorled pattern along with areas of fibrosis, findings characteristic of the classic histological presentation of DFSP (Figure 3a). Immunohistochemical staining showed strong positivity for CD34 (Figure 3b) further confirming the diagnosis.

**Figure 4:**
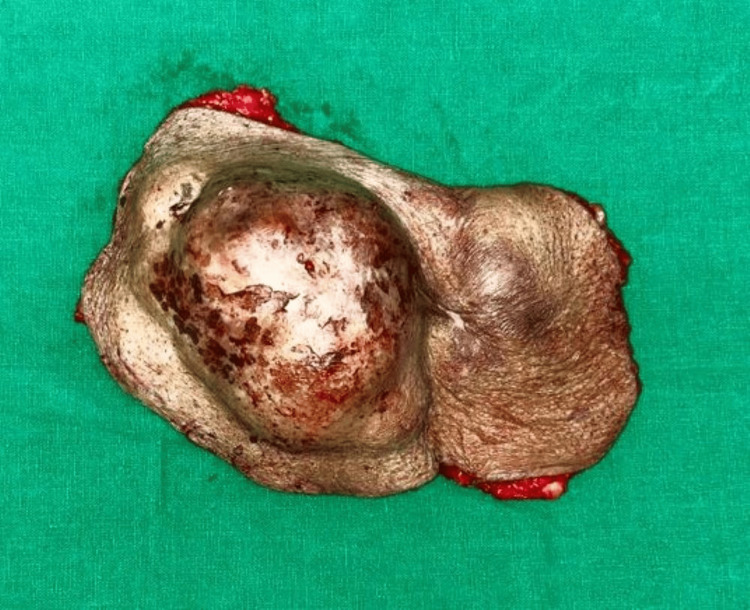
Excised specimen of DFSP DFSP - Dermatofibrosarcoma protuberans

Given the diagnosis of DFSP, the patient was planned and posted for wide local excision of the tumor. Under general anesthesia, a 2 cm margin of healthy tissue surrounding the tumor was excised, ensuring complete removal of the tumor (Figure 4).

**Figure 5:**
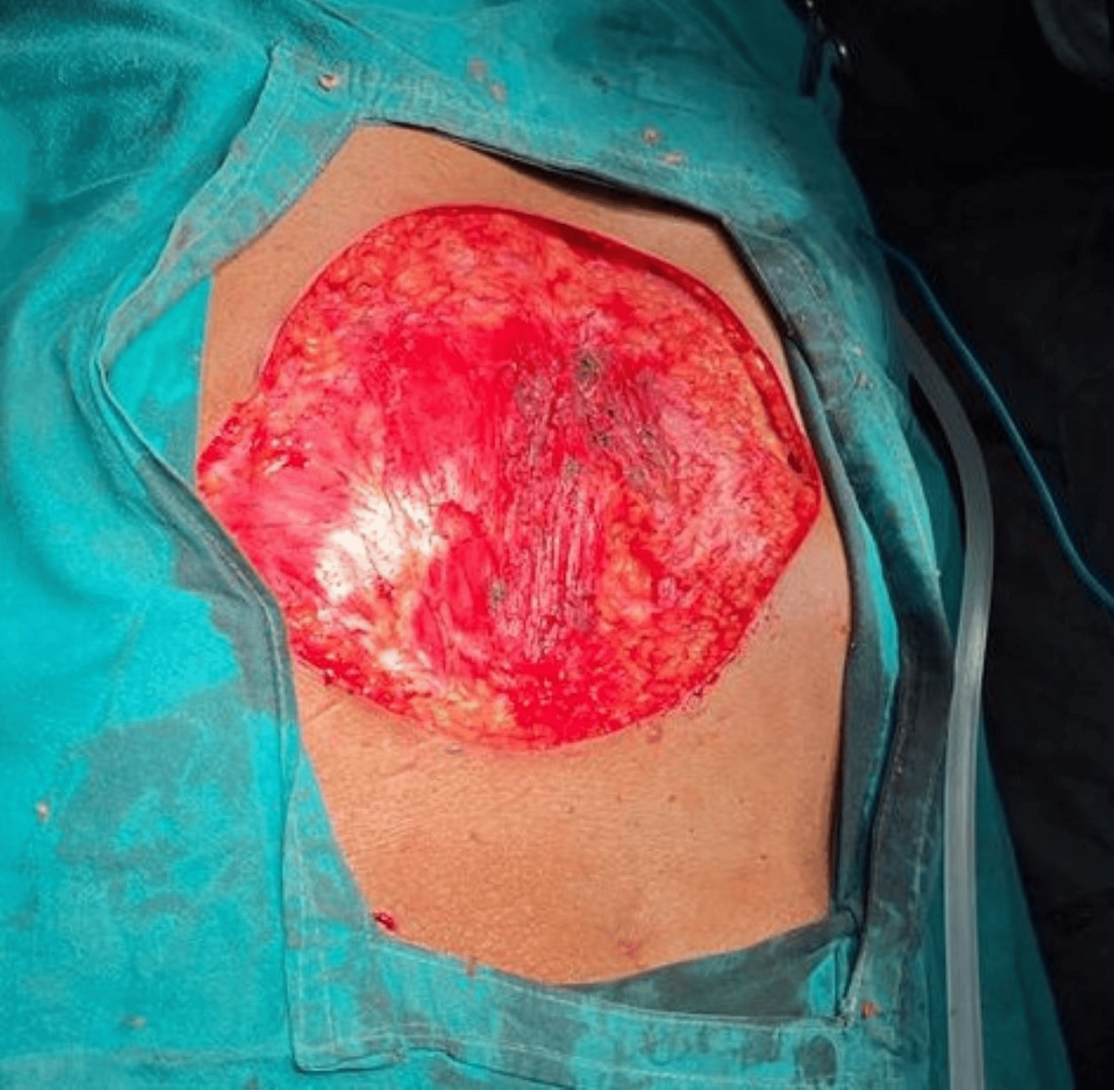
Intraoperative image of the anterior abdominal wall after complete excision of the tumor

Frozen section analysis of the surgical margins confirmed that no tumor cells remained. The resultant defect, measuring approximately 10 cm in diameter, extended to the fascia, necessitating reconstruction (Figure 5).

Due to the large size of the defect and the necessity to maintain abdominal wall integrity, a split-thickness skin graft was chosen for wound closure. The graft was harvested from the patient's left thigh and secured to the defect site. Dressings were applied, and the graft was bolstered with sterile dressing material to ensure proper adherence to the wound bed.

**Figure 6:**
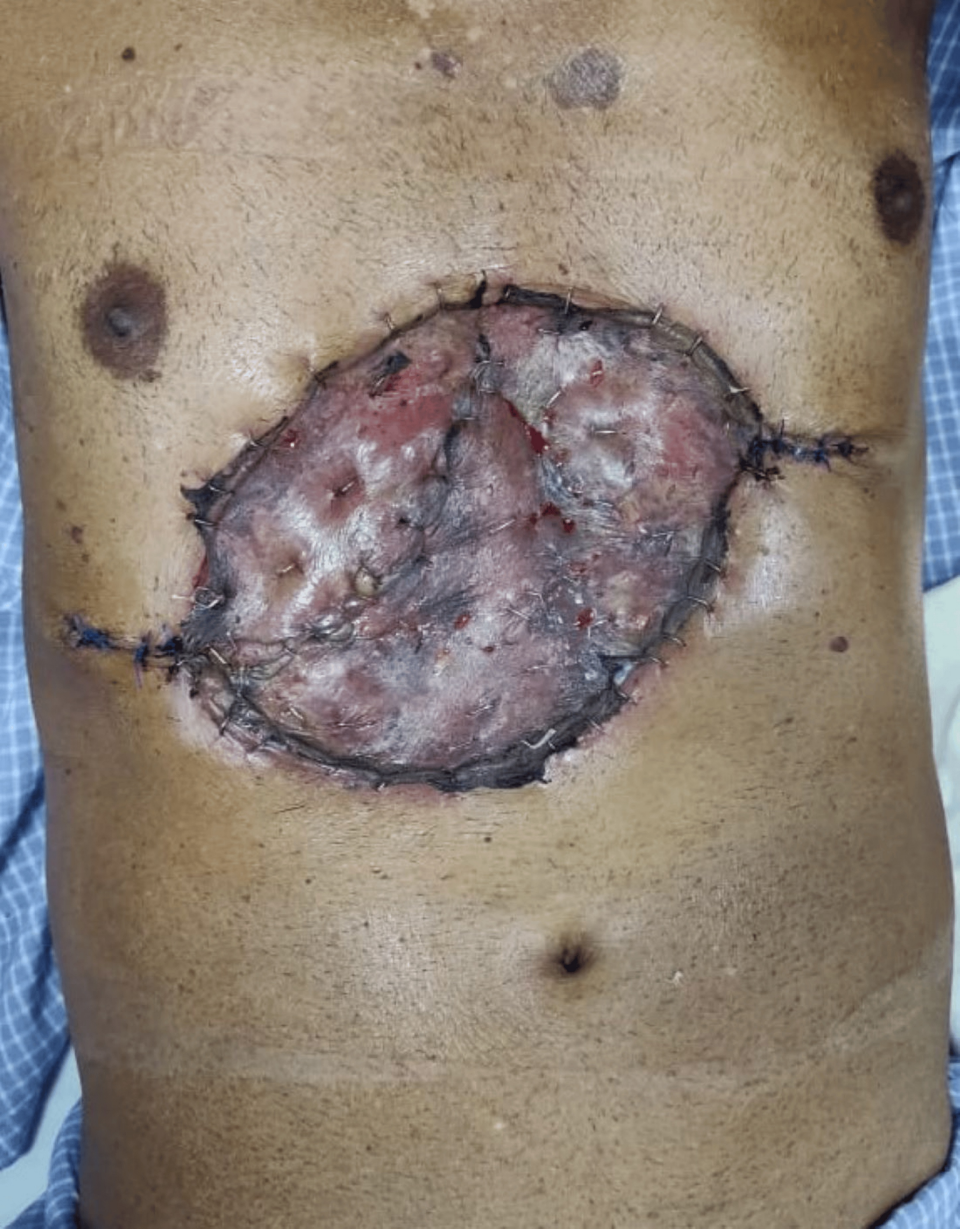
Clinical image of the local surgical site on post-operative day 10

The patient's post-surgery recovery was unremarkable, and he was discharged from the hospital on the 10th postoperative day after the surgical site was evaluated (Figure 6).

Over the subsequent nine months, the patient had continued regular follow-up visits without any evidence of tumor recurrence.

## Discussion

DFSP is a rare tumor that can originate from the dermal, subcutaneous, and in exceptional cases, muscular and fascial tissues. The precise underlying cause of this tumor remains elusive [5]. The most common predisposing factor is believed to be recurrent trauma to the skin at the site of the neoplasm. Additionally, pre-existing cutaneous scars and tattoos may increase the risk of developing this tumor [6].

DFSP can pose diagnostic challenges due to its rarity and overlapping features with other soft tissue tumors. Histopathology, particularly the storiform pattern and immunohistochemical analysis, is crucial for accurate diagnosis. CD34 positivity is a hallmark feature of DFSP, assisting in its distinction from other lesions [7].

Surgical excision with clear margins is the primary treatment for DFSP. The extent of excision is determined by clinical factors, including tumor size, depth, and proximity to vital structures. Wide local excision with a 2 cm margin of healthy tissue is often performed, and frozen section analysis of the surgical margins is critical to confirm clear margins [8]. Mohs micrographic surgery is an alternative surgical approach that aims to minimize the extent of tissue removal while ensuring a comprehensive assessment of the resection margins [9]. In our case, wide local excision was performed to achieve clear margins. The tumor was completely removed, and the defect extended to the fascia, necessitating reconstruction with a skin graft.

Reconstruction of the abdominal defect after DFSP excision poses a unique challenge, as it requires both functional and aesthetic restoration. Various reconstructive options, including local flaps, regional flaps, and skin grafts, may be considered. The selection of a reconstructive approach is contingent upon various factors, including the size and location of the surgical defect, as well as the patient's preferences and clinical needs. Skin grafts, such as split-thickness skin grafts, are commonly used for large, shallow defects. The advantages of skin grafting include simplicity, minimal donor site morbidity, and the ability to cover large areas. However, they may not be suitable for defects with deep fascial involvement or high-tension areas, as graft contracture can occur. In our case, a split-thickness skin graft was chosen due to the size of the defect and the need for an aesthetically pleasing result. Careful preoperative planning, donor site selection, and postoperative wound care are essential for successful graft take and optimal outcomes.

Diligent follow-up is paramount, as the risk of recurrence is greatest within the initial three-year period, necessitating routine evaluations every three to six months [2]. Metastasis is not very common. Increased age, specifically over 50 years, is associated with an elevated risk of local recurrence. Additionally, patients with certain demographic and tumor characteristics, such as Black race, male sex, lesions located on the head, neck, or leg, high mitotic activity, and increased histological cellularity, exhibit a greater risk of experiencing higher mortality rates [2]. Patients diagnosed with DFSP generally have a favorable prognosis, with a 10-year survival rate of approximately 99.1% [10].

## Conclusions

Dermatofibrosarcoma protuberans is an uncommon and locally aggressive soft tissue malignancy that exhibits a propensity for recurrence. Successful management of recurrent DFSP on the anterior abdominal wall involves wide local excision with clear margins and appropriate reconstruction. In this case, split-thickness skin grafting provided an effective solution for covering the surgical defect while preserving both form and function. Close long-term follow-up is crucial in cases of recurrent DFSP to monitor for any signs of local recurrence. The presented case emphasizes the importance of a multidisciplinary approach involving surgical oncologists, dermatologists, pathologists, and plastic surgeons to ensure optimal patient outcomes.
